# Transcriptome profile of goat folliculogenesis reveals the interaction of oocyte and granulosa cell in correlation with different fertility population

**DOI:** 10.1038/s41598-021-95215-z

**Published:** 2021-08-03

**Authors:** Shen Li, Junjie Wang, Hongfu Zhang, Dongxue Ma, Minghui Zhao, Na Li, Yuhao Men, Yuan Zhang, Huimin Chu, Chuzhao Lei, Wei Shen, Othman El-Mahdy Othman, Yong Zhao, Lingjiang Min

**Affiliations:** 1 College of Animal Sciences and Technology, Qingdao Agricultural University, Qingdao, 266109 People’s Republic of China; 2 College of Life Sciences, Qingdao Agricultural University, Qingdao, 266109 People’s Republic of China; 3 State Key Laboratory of Animal Nutrition, Institute of Animal Sciences, Chinese Academy of Agricultural Sciences, Beijing, 100193 People’s Republic of China; 4 Jining Animal Husbandry Development Center, Jining, People’s Republic of China; 5 Jining Agricultural Science Institute, Jining, People’s Republic of China; 6 Key Laboratory of Animal Genetics, Breeding and Reproduction of Shaanxi Province, College of Animal Science and Technology, Northwest A&F University, Yangling, 712100 People’s Republic of China; 7 Cell Biology Department, National Research Centre, Dokki, Giza, 12311 Egypt

**Keywords:** Cell biology, Computational biology and bioinformatics, Genetics, Physiology

## Abstract

To understand the molecular and genetic mechanisms related to the litter size in one species of two different populations (high litter size and low litter size), we performed RNA-seq for the oocytes and granulosa cells (GCs) at different developmental stages of follicle, and identified the interaction of genes from both sides of follicle (oocyte and GCs) and the ligand-receptor pairs from these two sides. Our data were very comprehensive to uncover the difference between these two populations regarding the folliculogenesis. First, we identified a set of potential genes in oocyte and GCs as the marker genes which can be used to determine the goat fertility capability and ovarian reserve ability. The data showed that *GRHPR*, *GPR84*, *CYB5A* and *ERAL1* were highly expressed in oocyte while *JUNB*, *SCN2A*, *MEGE8*, *ZEB2*, *EGR1*and *PRRC2A* were highly expressed in GCs. We found more functional genes were expressed in oocytes and GCs in high fertility group (HL) than that in low fertility group (LL). We uncovered that ligand-receptor pairs in *Notch* signaling pathway and transforming growth factor-β (*TGF*-β) superfamily pathways played important roles in goat folliculogenesis for the different fertility population. Moreover, we discovered that the correlations of the gene expression in oocytes and GCs at different stages in the two populations HL and LL were different, too. All the data reflected the gene expression landscape in oocytes and GCs which was correlated well with the fertility capability.

## Introduction

The bidirectional interactions of oocytes and surrounding granulosa cells (GCs) play vital roles in folliculogenesis which is crucial for the oocyte to acquire the developmental competence. During folliculogenesis, the cellular communication between oocytes and GCs is intricate because both oocytes and GCs pose active regulatory functions. The surrounding GCs provide cAMP^1^, calcium^2^, some metabolites^3,4^ and many unknown signals^5^ to support the meiotic process and cytoplasmic maturation of oocytes. On the other hand, the oocyte secretes many factors such as growth factors to stimulate the differentiation and proliferation of GCs^3^. Moreover, RNAs can be transferred from one side to the other side to be involved in the folliculogenesis^6^. However, the specific mechanisms of transporting these macromolecules are unknown.

Recently, the impressive body of data suggested that the transcriptome profile of GCs^7–17^ and oocytes^18–21^ reflects the developmental potential of follicles and even the following successful fertilization and embryo formation. Furthermore, it has been reported that the dynamic transcriptional regulation in both oocyte and GCs plays very important roles in the follicle growth and oocyte maturation^22^. Late on, Zhang et al. (2018) suggested that the key event to understanding the molecular interactions that regulates oocyte maturation and follicle growth is to characterize the transcriptome of oocyte and GC at different developmental stages^23^. Moreover, Biase and Kimble found that the bidirectional communication between oocyte and GCs is very complicated in which ligand-receptor pairs in these two sides play important roles in the regulation of the expression of thousands of genes^24^. At the same time the interactions of ligand-receptors are also important for the acquisition of oocyte developmental competence.

The domestic goats (Capra hircus) are widely raised as livestock throughout the world for meat, milk, skin and fiber. Moreover, recently goats have been applied in biomedical research^25–27^. Therefore, the increase in the population of goats has been a strong enthusiasm in which the improvement of reproductive traits especially the litter size has been of expanding enthusiasm for goats^28,29^ However, a number of factors affect litter size and ovulation rate is the key one. Although a few studies have analyzed the gene expression of oocyte or GCs in goat ovaries, the data are not complete because they only determined the gene expression in one population, or at one developmental stage, or just oocyte or just GCs. To understand the molecular and genetic mechanisms related to the litter size in one species of two different populations (high litter size and low litter size), we performed RNA-seq for the oocytes and GCs at different developmental stages of follicle, and identified the interaction of genes from both sides of follicle (oocyte and GCs) and the ligand-receptor pairs from these two sides. Our data are very comprehensive to uncover the difference between these two populations regarding the folliculogenesis.

## Methods

### Study design

This study was approved by the Committee on the Ethics of Animal Experiments of Qingdao Agricultural University IACUC (Institutional Animal Care and Use Committee) in strict accordance with the recommendations in the Guide for the Care and Use of Laboratory Animals of the National Institutes of Health^30,31^*.*

Ji’Ning grey goat is one of Chinese oldest domesticated goat species with high fecundity^25^. In current study, Ji’Ning grey goats were used. 15 goats from High fertility group (≥ 3/litter; HL) and 15 goats from Low fertility group (≤ 2/litter; LL). All the ovarian follicles (small: < 3 mm; medium: 3–7 mm; large: ˃ 7 mm; small represents early stage while medium and large represent late stage as discussed in the result section) from each group were collected. Then oocytes and GCs were mechanically separated by pipet a few times. The oocytes from small, medium, and large follicles of High fertility group were labeled as HSO, HMO and HLO respectively. The oocytes from small, medium, and large follicles of Low fertility group were labeled as LSO, LMO and LLO respectively. The GCs from small, medium, and large follicles of High fertility group were labeled as HSG, HMG and HLG respectively. The GCs from small, medium, and large follicles of Low fertility group were labeled as LSG, LMG and LLG respectively. All the oocytes from small, medium, and large follicles of High fertility group were pulled together respectively. Then the oocytes from each stage was separated into different samples (five samples for each stage, LSO1, LSO2, LSO3, LSO4, LSO5; LMO1, LMO2, LMO3, LMO4, LMO5; LLO1, LLO2, LLO3, LLO4, LLO5; HSO1, HSO2, HSO3, HSO4, HSO5; HMO1, HMO2, HMO3, HMO4, HMO5; HLO1, HLO2, HLO3, HLO4, HLO5; 2–3 oocytes/each sample). And the GCs from each stage was separated into different samples (five samples for each stage, LSG1, LSG2, LSG3, LSG4, LSG5; LMG1, LMG2, LMG3, LMG4, LMG5; LLG1, LLG2, LLG3, LLG4, LLG5; HSG1, HSG2, HSG3, HSG4, HSG5; HMG1, HMG2, HMG3, HMG4, HMG5; HLG1, HLG2, HLG3, HLG4, HLG5; 10–20 GCs/each sample). Then the samples were subjected to next steps. The goat ovaries were purchased from the Jining Castle Peak Sheep Farm Co., Shandong, P.R. China. This study is reported in accordance with ARRIVE guidelines.

### Library preparation, sequencing and data analysis

#### Library preparation and sequencing

We prepared libraries for the oocytes and GCs separately. For GCs, total RNA was extracted as described in our early articles^30^. Then Ribo-Zero rRNA Removal Kit was used to remove rRNA to purify the RNA samples. Then Ribo-Zero rRNA Removal Kit and DNA polymerase I and RNaseH were used to make the double strands cDNA followed by the sequencing by Illumina HiSeq X Ten equipment following the manufacturer’s instruction. For the oocytes, total RNA was isolated as described in our early article^30^. Then the mRNA was enriched by the Oligo (dT) beads. Then the mRNA samples were used to make the double strand cDNA templates for the sequencing by Illumina HiSeq X Ten equipment following the manufacturer’s instruction.

#### Gene expression levels and principal component analysis

The libraries were aligned against the Capra_hircus genome^31^. Only reads showing a unique match to the genome and less than five mismatches were further filtered to eliminate duplicates with picard. The bam files containing non-duplicated reads were employed as input for Cufflinks (v.2.2.1), together with Ensembl gene annotation, to obtain FPKM (Fragments Per Kilobase of exon model per Million mapped fragments) data. Genes were subjected to analytical procedures if FPKM > 0.5. All statistical analyses were conducted in R software (Biase FH, Kimble 2018). To identify the main sources of variation in the dataset, we employed the FPKM values as the input for principal component analysis using the FactorMiner R package. The significance of the principal components was obtained with the Seurat package via a permutation test, after 1000 randomized samplings^24^.

#### Identification of putative ligands and receptors in cumulus-oocyte complexes

First, we produced a comprehensive protein–protein interaction (PPI) database as described in the articles^24,32–34^. Briefly, the gene from our data were used to search the ligand-receptor pairs as reported in these articles. Then the ligand-receptor pairs from GCs or oocytes were mapped the gene identifier^24^.

#### Calculation of the pairwise correlation of transcript levels between genes expressed in oocytes and surrounding GCs

We calculated the Spearman’s correlation coefficient^30^ between the expression levels of genes expressed in oocytes and the surrounding GCs. Then the data were expressed by heatmap package in R stadio.

#### Functional annotation

The function of the different expressed genes was enriched by Metascape online.

### q-RT-PCR

The procedure for mRNA q-RT-PCR was reported in our early publications using qPCR Master Mix (Roche, German) by the Roche LightCycler1 480 (Roche, German)^31^. The primers for qPCR analysis were synthesized by Invitrogen and present in Table S1. Briefly, total RNA was extracted using TRIzol Reagent (Invitrogen Corp., Carlsbad, CA, USA) and purified using an RT2 qPCR-Grade RNA Isolation Kit from SABiosciences Co., Ltd (MD, USA). Total RNA was quantified using a Nanodrop 3300 (ThermoScientific, DE, USA). The quality of RNA was controlled by the A260:A280 ratio being greater than 2.0 and confirmed by electrophoreses, with a fraction of each total RNA sample with sharp 18S and 28S ribosomal RNA (rRNA) bands as reported in our recent publication. One microgram of total RNA was used to make the first strand cDNA in 20 μl. The program for the reaction of miRNA and lncRNA was 25 °C for 30 min, 42 °C for 30 min, 85 °C for 5 min, then 4 °C or on ice. The qPCR was performed with the Roche LightCycler 480 (Roche, Germany) and the reaction was as follows: Step 1, 95 °C for 3 min; Step 2, 40 cycles of 95 °C for 12 s; 62 °C for 40 s. Three independent experimental samples were analyzed.

### Immunohistofluorescence staining (IHF)

The procedure for immunofluorescence staining is reported in our recent publication^30,31^. Briefly, goat ovarian Sects. (5 μm; paraffin embedded) were prepared and subjected to antigen retrieval. Sections were then blocked with normal goat serum in phosphate buffer saline (PBS), followed by incubation (1:150 in PBS-1% BSA) with primary antibodies (GPD1, Cat. #: bs-2388R; TST, Cat. #: bs-19187R; both from Beijing Biosynthesis Biotechnology CO.) at 4 °C overnight. After a brief wash, sections were incubated with goat anti-rabbit or donkey anti-goat secondary Abs (1:100 in PBS; Beyotime Institute of Biotechnology, Shanghai, P.R. China) at RT for 30 min and then counterstained with 4′,6-diamidino-2-phenylindole (DAPI). The stained sections were visualized using a Nikon Eclipse TE2000-U fluorescence microscope (Nikon, Inc., Melville, NY), and the captured fluorescence images were analyzed using MetaMorph software.

### Ethics approval and consent to participate

This study was approved by the Committee on the Ethics of Animal Experiments of Qingdao Agricultural University IACUC (Institutional Animal Care and Use Committee) in strict accordance with the recommendations in the Guide for the Care and Use of Laboratory Animals of the National Institutes of Health.

## Results

### The transcript profiles of oocytes and granulosa cells during folliculogenesis

**Figure 1:**
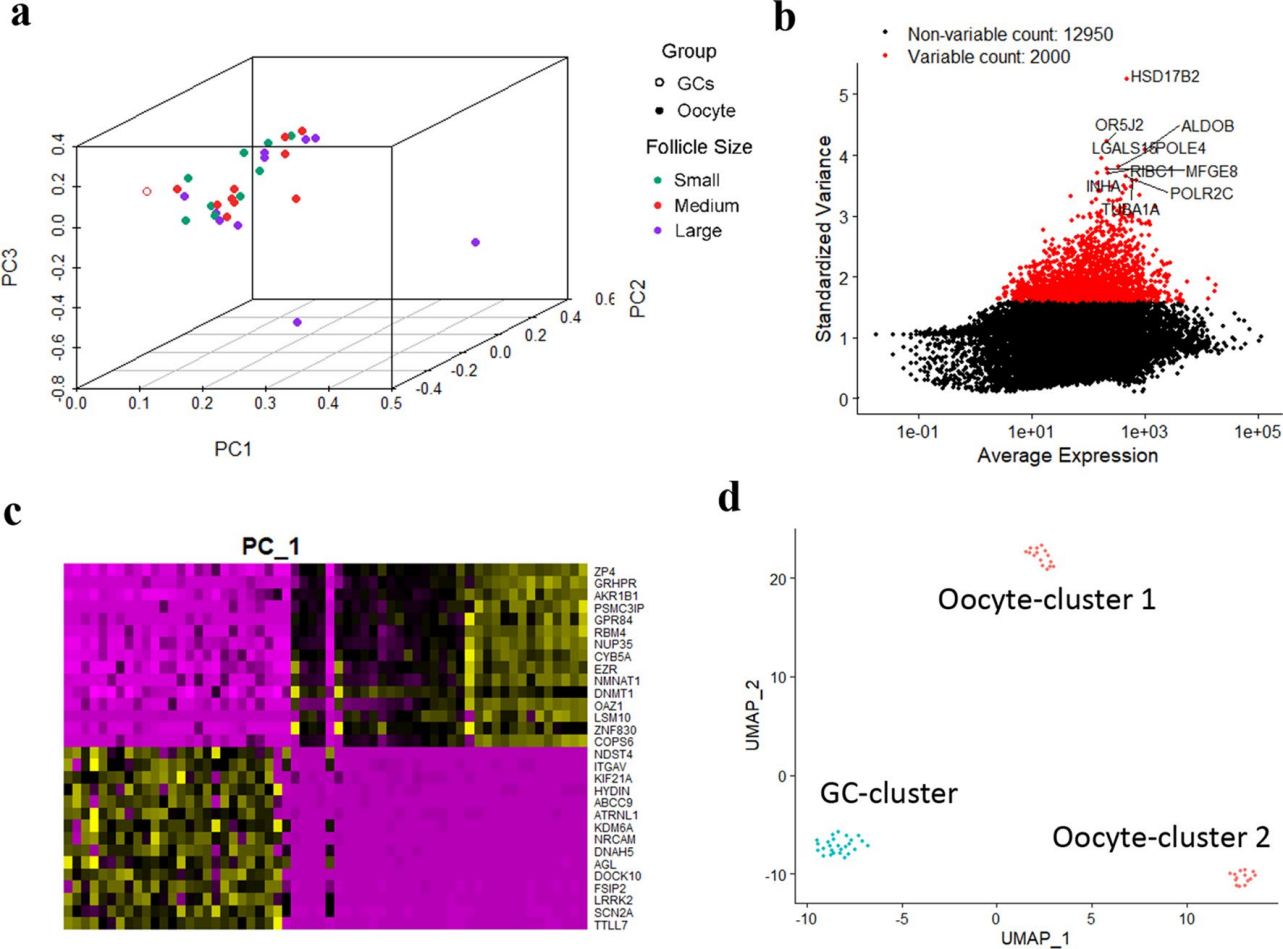
The global transcriptomic character of oocytes and granulosa cells in Jining grey goat. (**a**) 3D PCA plot of oocyte and granulosa cell (GCs) samples. (**b**) Highly variable genes of samples identified by Seurat (top 10 with label). (**c**) Genes in PC1 identified by Seurat. (**d**) UMAP cluster of oocyte and granulosa cells.

In order to discover the interactions of GCs and oocytes in the folliculogenesis, we analyzed the gene expression in different stages of oocytes and GCs of different fertility populations (HL, LL). We found that during the development, the gene expression profiles changed dramatically in the different stages of oocytes (Fig. 1a, Tables S2, S3). The gene expression profiles for different stages of GCs did not change much (Fig. 1a). In order to discover the molecular difference in oocytes and GCs, we used Seurat program to detect the marker genes and found the highly changed genes in oocytes and GCs, such as *HSD17B2* and *INHA* (Fig. 1b). Moreover, the PCA analysis discovered that important marker genes in oocytes and GCs in PC1 component (Fig. 1c). The cluster analysis by UMAP program found that GCs and oocytes can be separated very well (Fig. 1d). However, there were two subclusters of oocytes (Fig. 1d) which indicated that there was some difference for gene expression in different stages of oocytes.

**Figure 2:**
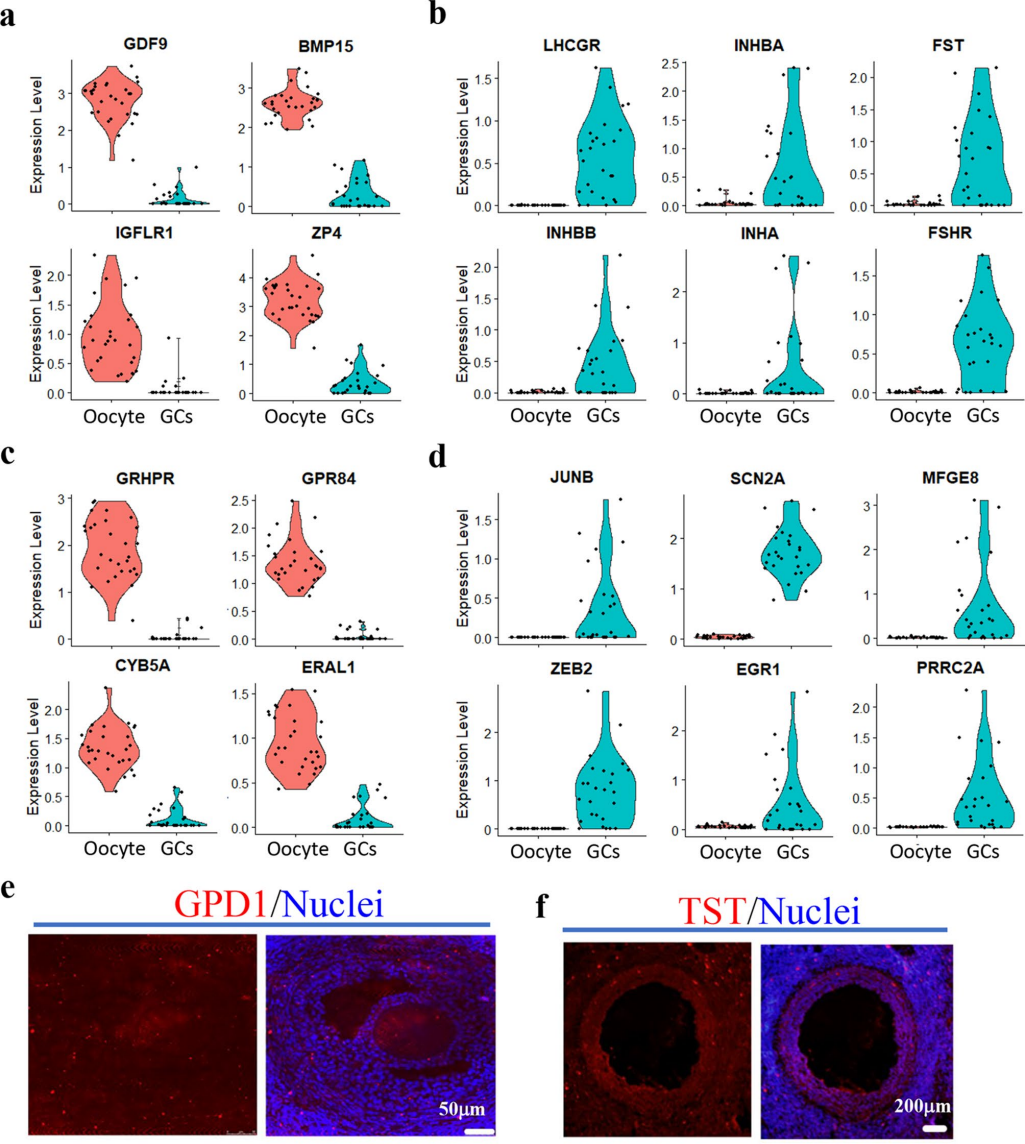
The molecular marker genes of oocytes and granulosa cells in Jining grey goat. (**a**) Typical marker genes in oocyte. (**b**) Typical marker genes in GCs. (**c**) Novel identified marker genes in oocytes. (**d**) Novel identified marker genes in GCs. (**e**) IHF for GPD1. (**f**) IHF for TST.

Based on the significant expressed marker genes, we found lots of dramatically different expressed genes in oocytes or GCs (Fig. 2), such as *GDF9* and *BMP15* which were highly expressed in oocytes while *FST*, *FSHR*, and *LHCGR* which were highly expressed in GCs (Fig. 2a,b). At the same time, we discovered new marker genes which can be used to separate the different cell types, such as *GRHPR*, *GPR84*, *CYB5A*, *ERAL1* for oocytes (Fig. 2c), while *JUNB*, *SCN2A*, *MEGE8*, *ZEB2*, *EGR1*, and *PRRC2A* for GCs (Fig. 2d). These genes can be potential marker genes of goat follicular cells. At the same time, the protein of the different genes for oocytes (*GPD1*) and GCs (*TST*) were analyzed by IHF staining (Fig. 2e,f).

### Dynamic gene expression profiles in different stages of oocytes of the two fertility populations

There were two population of goats used in this investigation: high fertility group (> 3/litter; HL) and low fertility group (≤ 2/litter; LL). The main purpose of this investigation was to explore the difference in the gene expression in different stages (large follicle > 7 mm in size; medium follicle 3–7 mm in size; small follicle < 3 mm in size) of oocytes and GCs in the two fertility population goats.

**Figure 3:**
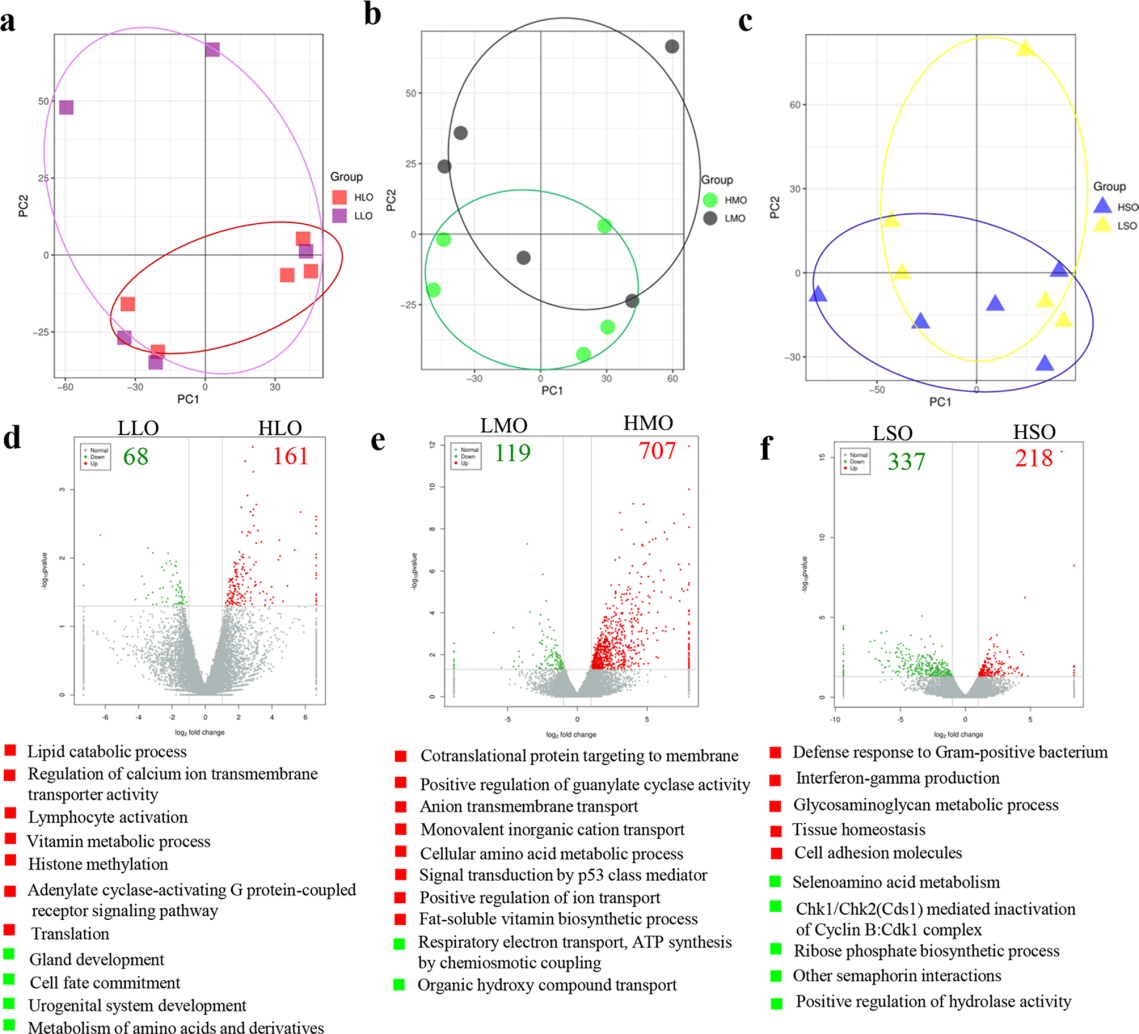
Dynamic gene expression in oocytes of different fertility groups throughout folliculogenesis. (**a**) PCA of oocytes in large follicles of high fertility and low fertility samples (HLO vs. LLO). (**b**) PCA of oocytes in medium follicles of high fertility and low fertility (HMO vs. LMO). (**c**) PCA of oocytes in small follicles of high fertility and low fertility (HSO vs. LSO). (**d**) Differentially expressed genes (DEGs) in oocytes of large follicles from high fertility group and low fertility group, and the top enriched function pathways. Red indicates the gene expressed higher in HLO; green indicate the gene expressed higher in LLO. (**e**) Differentially expressed genes (DEGs) in oocytes of medium follicles from high fertility group and low fertility group, and the top enriched function pathways. Red indicates the gene expressed higher in HMO; green indicate the gene expressed higher in LMO. (**f**) Differentially expressed genes (DEGs) in oocytes of small follicles from high fertility group and low fertility group, and the top enriched function pathways. Red indicates the gene expressed higher in HSO; green indicate the gene expressed higher in LSO.

PCA analysis found that the gene expression pattern was closer for the oocytes in large follicles in high fertility group (HLO) than that in low fertility group (LLO) (Fig. 3a). Similarly, the gene expression pattern was closer for the oocytes in medium follicles in high fertility group (HMO) than that in low fertility group (LMO) (Fig. 3b). Moreover, the gene expression pattern was closer for the oocytes in small follicles in high fertility group (HSO) than that in low fertility group (LSO) (Fig. 3c). The data suggested that the oocytes in high fertility group were more homogeneous than that in low fertility group.

There were 161 genes highly expressed in HLO while 68 genes were highly expressed in LLO (Fig. 3d). The functions of the changed genes were enriched by Metascape online. Most of the enriched functional pathways were related to metabolism which was related to the growth of large follicle to prepare for ovulation (Fig. 3d).

There were 707 genes highly expressed in HMO while 119 genes were highly expressed in LMO (Fig. 3e). Most of the enriched functional pathways were related to transport which was related to the interaction of oocytes and GCs for the exchange of materials (Fig. 3e).

There were 218 genes highly expressed in HSO while 337 genes were highly expressed in LSO (Fig. 3f). The enriched functional pathways were in wide range of molecular functions. There were some pathways related to cell defense in the increased genes (Fig. 3f).

The data in this section suggested that for the gene expression, the big difference was in the oocytes of the medium follicles. And the HMO has the highly developmental potential to HLO.

### Dynamic gene expression profiles in different stages of GCs of the two fertility populations

**Figure 4:**
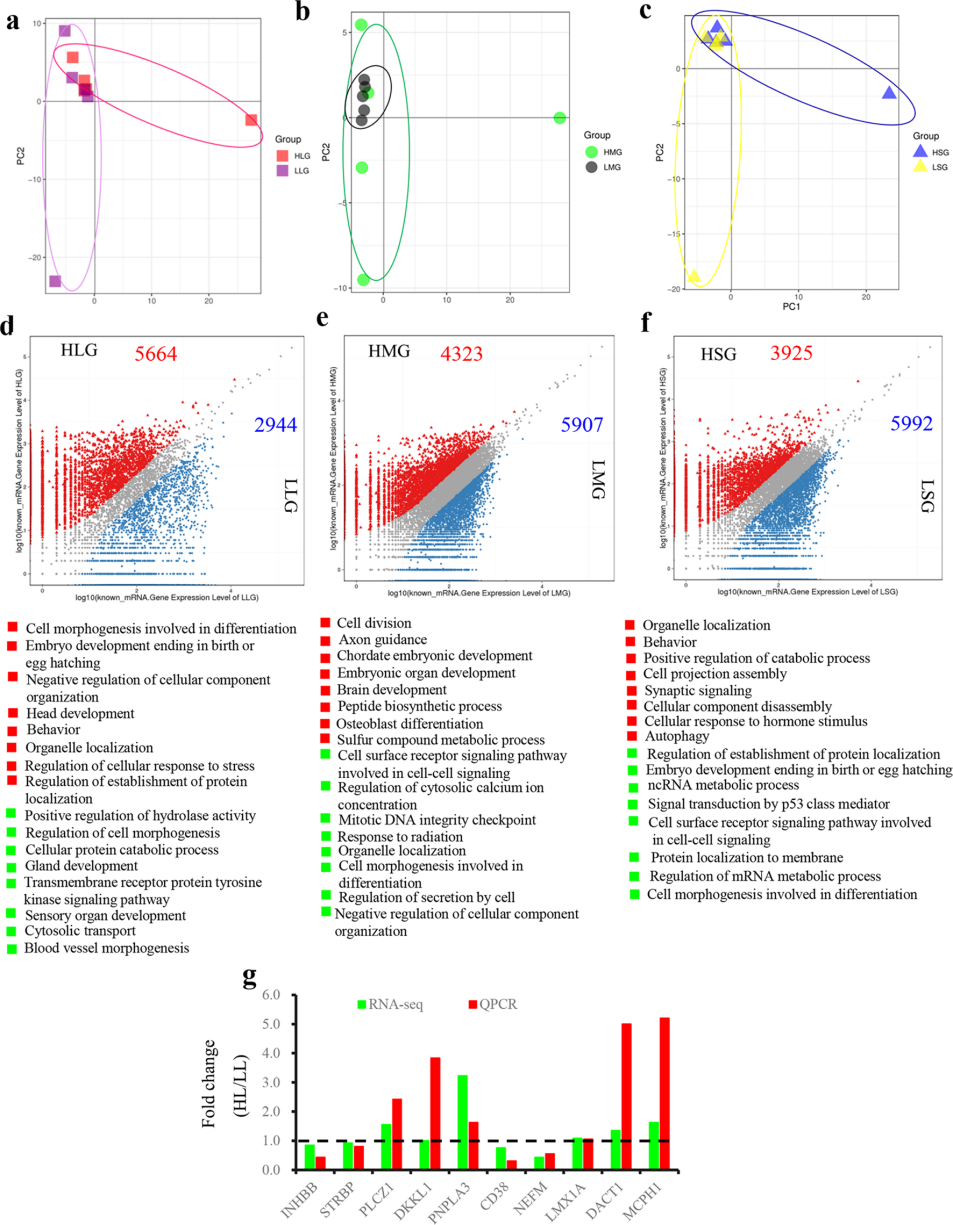
Dynamic gene expression in GCs of different fertility groups throughout folliculogenesis. (**a**) PCA of GCs in large follicles of high fertility and low fertility samples (HLG vs. LLG). (**b**) PCA of GCs in medium follicles of high fertility and low fertility (HMG vs. LMG). (**c**) PCA of GCs in small follicles of high fertility and low fertility (HSG vs. LSG). (**d**) Differentially expressed genes (DEGs) in GCs of large follicles from high fertility group and low fertility group, and the top enriched function pathways. Red indicates the gene expressed higher in HLG; green indicate the gene expressed higher in LLG. (**e**) Differentially expressed genes (DEGs) in GCs of medium follicles from high fertility group and low fertility group, and the top enriched function pathways. Red indicates the gene expressed higher in HMG; green indicate the gene expressed higher in LMG. (**f**) Differentially expressed genes (DEGs) in GCs of small follicles from high fertility group and low fertility group, and the top enriched function pathways. Red indicates the gene expressed higher in HSG; green indicate the gene expressed higher in LSG. (**g**) The comparation of RNA-seq data and PCR data for GCs. Fold change of high fertility group compared to low fertility group.

PCA analysis uncovered that the gene expression pattern was similar for the GCs in large follicles of high fertility group (HLG) and low fertility group (LLG) (Fig. 4a); in medium follicles of high fertility group (HMG) and low fertility group (LMG) (Fig. 4b); in small follicles of high fertility group (HSG) and low fertility group (LSG) (Fig. 4c).

There were 5664 genes highly expressed in HLG while 2944 genes were highly expressed in LLG (Fig. 4d). There were two pathways related to differentiation and embryo development were enriched in the increased genes in HLG (Fig. 4d).

There were 4323 genes were highly expressed in HMG while 5907 genes were highly expressed in LMG (Fig. 4e). There were some pathways related to cell division, and embryonic development enriched in the increased genes in HMG (Fig. 4e) which further indicated that GCs in the large follicles of high fertility group pose more potential for the oocyte development.

There were 3925 genes were highly expressed in HSG while 5992 genes were highly expressed in LSG (Fig. 4f). The enriched functional pathways were diverse in this comparation (Fig. 4f) which suggested that at small follicle stage, the GCs in high fertility and low fertility groups are close.

Some of the gene expression levels were confirmed by q-RT-PCR (Fig. 4g).

### Road map of ligands and receptors between oocytes and GCs in the two goat populations

The ligands and receptors play vital roles in the folliculogenesis, especially the interaction between the oocyte and GCs is bidirectional which is mediated by ligands and receptors interaction^24,32–34^. We determined the expression of genes encoding ligand-receptor pairs in oocytes and GCs.

**Figure 5:**
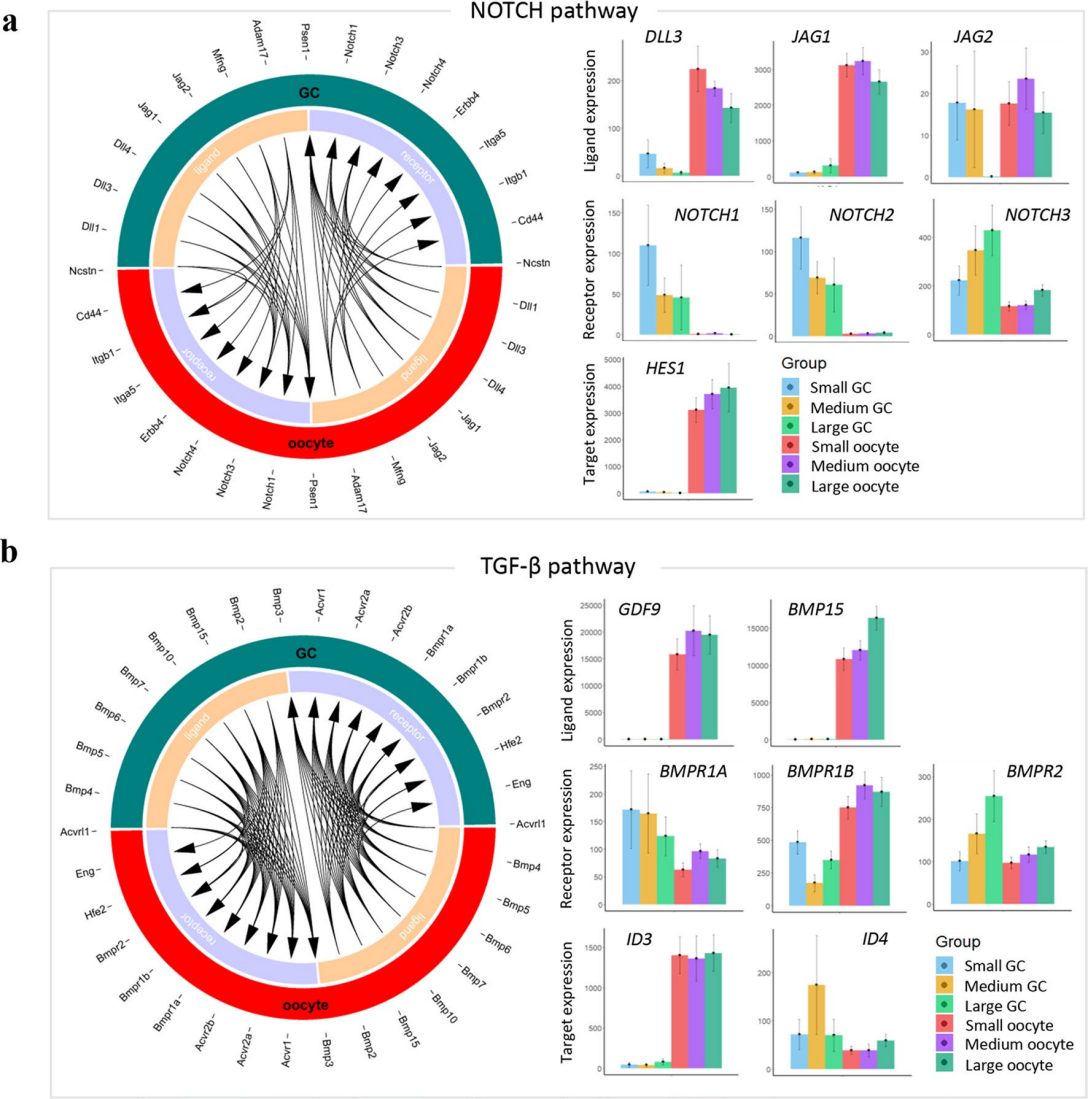
Ligand–receptor pairs in oocytes and GCs. (**a**) Ligand and receptor encoding genes in NOTCH pathway expressed in goat follicular oocyte and GCs. Left, outside circle showed in GC or oocyte; while inside circle showed ligands or receptors. Right, relative expression. (**b**) Ligand and receptor encoding genes in TGF-β pathway expressed in goat follicular oocyte and GCs. Left, outside circle showed in GC or oocyte; while inside circle showed ligands or receptors. Right, relative expression. Free R package Circlize (version0.4.11; https://cran.r-project.org/web/packages/circlize/) software was used.

*NOTCH* signaling pathway and *TGF*-β signaling pathway are the two most important signaling pathways in the folliculogenesis, and we found that many interesting unique protein–protein interactions (PPIs) of ligands and receptors for the oocytes and GCs. In *NOTCH* signaling pathway, the ligands *DLL3* and *JAG1* were specifically highly expressed in oocytes, while the ligand *JAG2* was highly expressed in early stages of GCs and all stages of oocytes (Fig. 5a). The receptors *NOTCH1*, *NOTCH2* were highly expressed in GCs while *NOTCH3* was expressed in oocytes and GCs. Especially, *NOTCH1* and *NOTCH2* were very highly expressed in early stages of GCs (Fig. 5a), while *NOTCH3* was very highly expressed in late stages of GCs (Fig. 5a). And the target gene *HES1* was specifically expressed in oocytes (Fig. 5a).

In *TGF*-β signaling pathway, the ligands *GDF9* and *BMP15* were specifically expressed in oocytes, while their receptors *BMPR1A*, *BMPR1B* and *BMPR2* were expressed in oocytes and GCs while the expression was time dependent (developmental stages) (Fig. 5b). The target genes *ID3* was expressed in oocytes and *ID4* was expressed in GCs and oocytes (Fig. 5b).

Cell–cell junctions also are very important for oocyte-GCs communication. The junction protein *GJC1* and *GJA4* were highly expressed in oocytes while *GJA1* and *GJA9* were highly expressed in GCs (Fig. S1).

**Figure 6:**
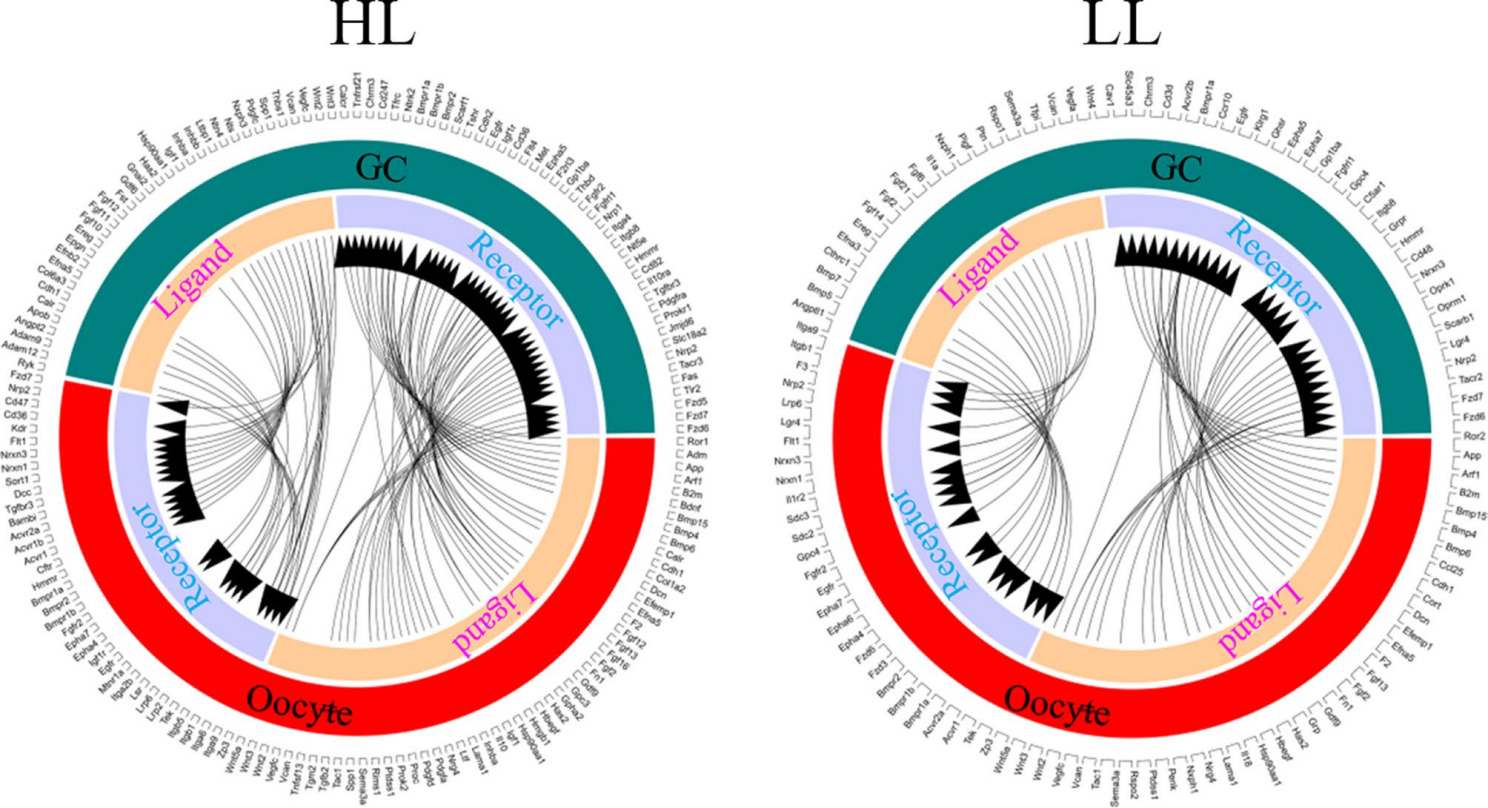
Ligand–receptor pairs, and ligand and receptor encoding genes expressed in high fertility and low fertility groups oocyte and GCs of goat large follicles. Outside circle showed in GC or oocyte; while inside circle showed ligands or receptors. Free R package Circlize (version0.4.11; https://cran.r-project.org/web/packages/circlize/) software was used.

**Figure 7:**
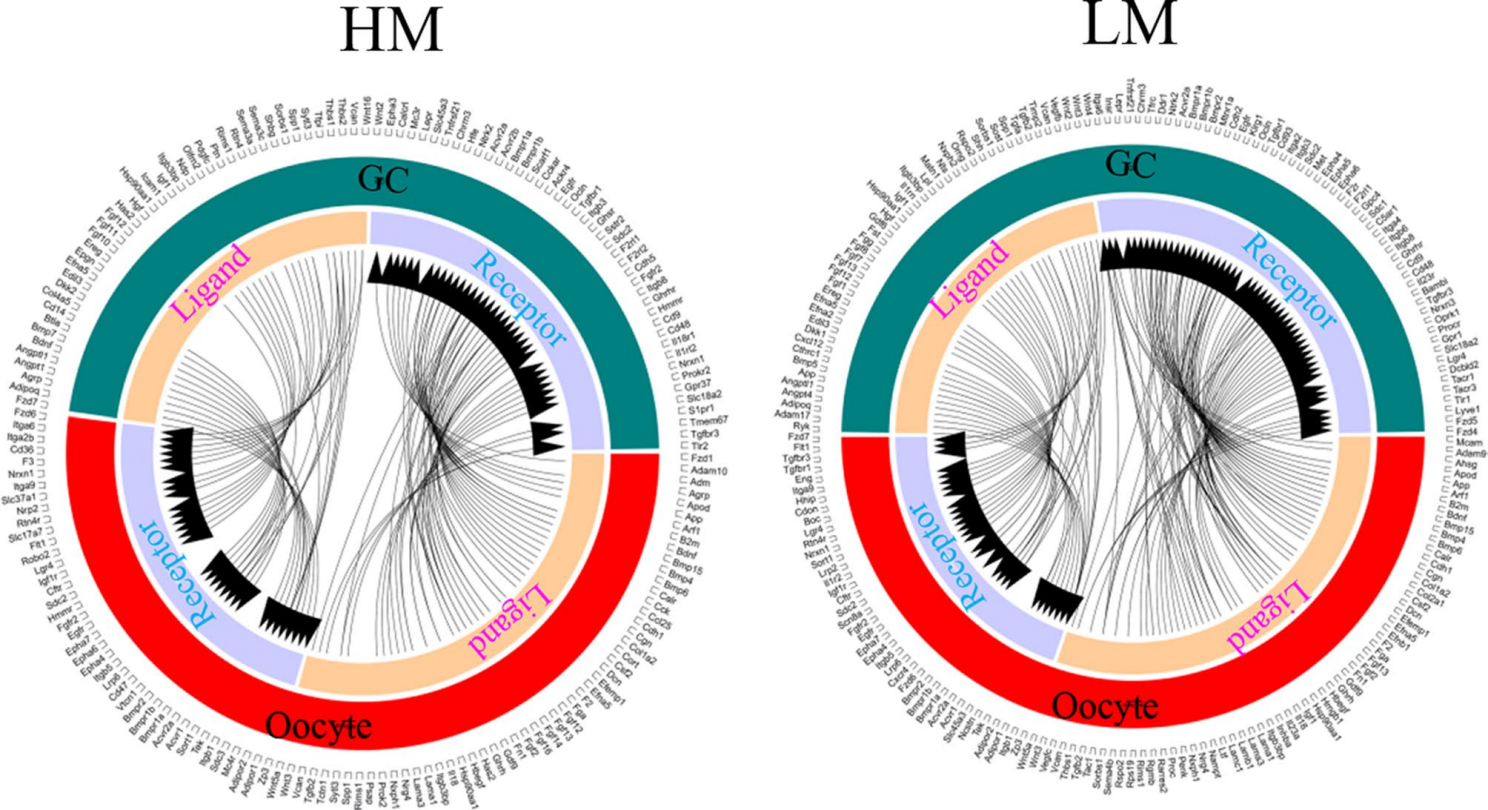
Ligand–receptor pairs, and ligand and receptor encoding genes expressed in high fertility and low fertility groups oocyte and GCs of goat medium follicles. Outside circle showed in GC or oocyte; while inside circle showed ligands or receptors. Free R package Circlize (version0.4.11; https://cran.r-project.org/web/packages/circlize/) software was used.

**Figure 8:**
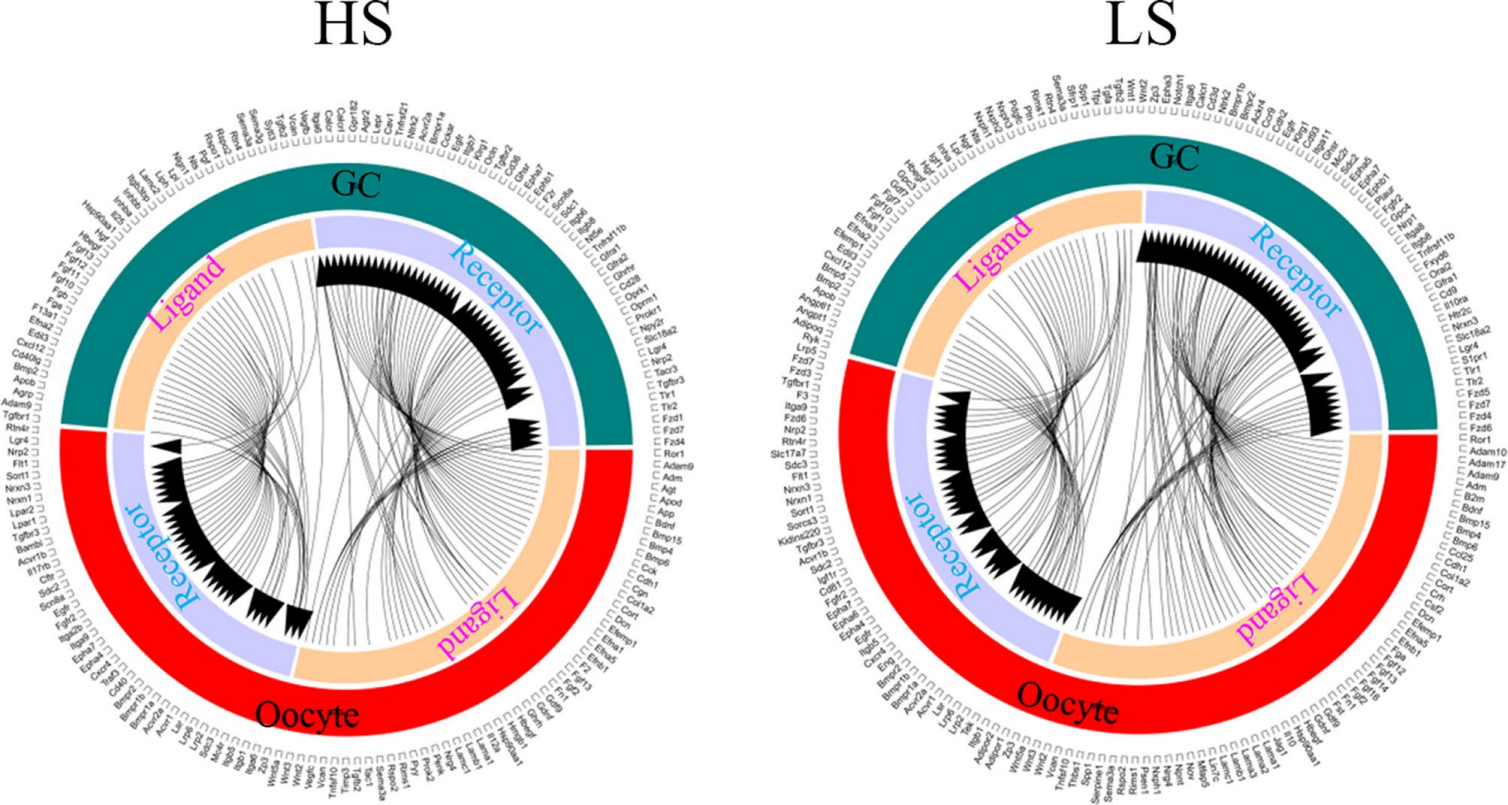
Ligand–receptor pairs, and ligand and receptor encoding genes expressed in high fertility and low fertility groups oocyte and GCs of goat small follicles. Outside circle showed in GC or oocyte; while inside circle showed ligands or receptors. Free R package Circlize (version0.4.11; https://cran.r-project.org/web/packages/circlize/) software was used.

Moreover, based on the gene expression the ligands and receptors pairs have been identified in different stages of oocytes and GCs of the two population goats (Figs. 6, 7, 8). 44 ligand-receptor pairs have been identified for the ligands in GCs and receptors in oocytes, while 65 ligand-receptor pairs have been found for the ligands in oocytes and receptors in GCs in the large follicles of high fertility group (HL) (Fig. 6). 27 ligand-receptor pairs have been identified for the ligands in GCs and receptors in oocytes, while 42 ligand-receptor pairs have been found for the ligands in oocytes and receptors in GCs in the large follicles of low fertility group (LL) (Fig. 6). 47 ligand-receptor pairs have been identified for the ligands in GCs and receptors in oocytes, while 68 ligand-receptor pairs have been found for the ligands in oocytes and receptors in GCs in the medium follicles of high fertility group (HM) (Fig. 7). 50 ligand-receptor pairs have been identified for the ligands in GCs and receptors in oocytes, while 97 ligand-receptor pairs have been found for the ligands in oocytes and receptors in GCs in the medium follicles of low fertility group (LM) (Fig. 7). 50 ligand-receptor pairs have been identified for the ligands in GCs and receptors in oocytes, while 69 ligand-receptor pairs have been found for the ligands in oocytes and receptors in GCs in the small follicles of high fertility group (HS) (Fig. 8). 55 ligand-receptor pairs have been identified for the ligands in GCs and receptors in oocytes, while 82 ligand-receptor pairs have been found for the ligands in oocytes and receptors in GCs in the small follicles of low fertility group (LS) (Fig. 8).

The functions of these PPI have been enriched by Metascape online. There were some specific functional pathways in the different stages of follicles of high or low fertility population. The interesting pathways “Signaling pathways regulating pluripotency of stem cells” and “*TGF*-beta signaling pathway” were enriched in the HL group not in LL population (Fig. S2). The pathways “developmental growth” and “epithelial cell differentiatio” were enriched in the HM group not in LM population (Fig. S3). The pathways “developmental growth”, “*MAPK* cascade”, “ECM-receptor interaction” and “GPCR ligand binding” were enriched in the HS group not in LS population (Fig. S4). The data suggested that the ligand-receptor interactions were different in the two fertility population which may be related to the fertility capability in goat.

### Co-regulated gene expression between the oocyte and GCs of the two fertility populations

The analysis of the gene co-expression between oocytes and GCs in different stages of follicles in two different fertility populations was performed in current investigation to explore the difference in the interactions of oocytes and GCs at different stages and in different populations.

**Figure 9:**
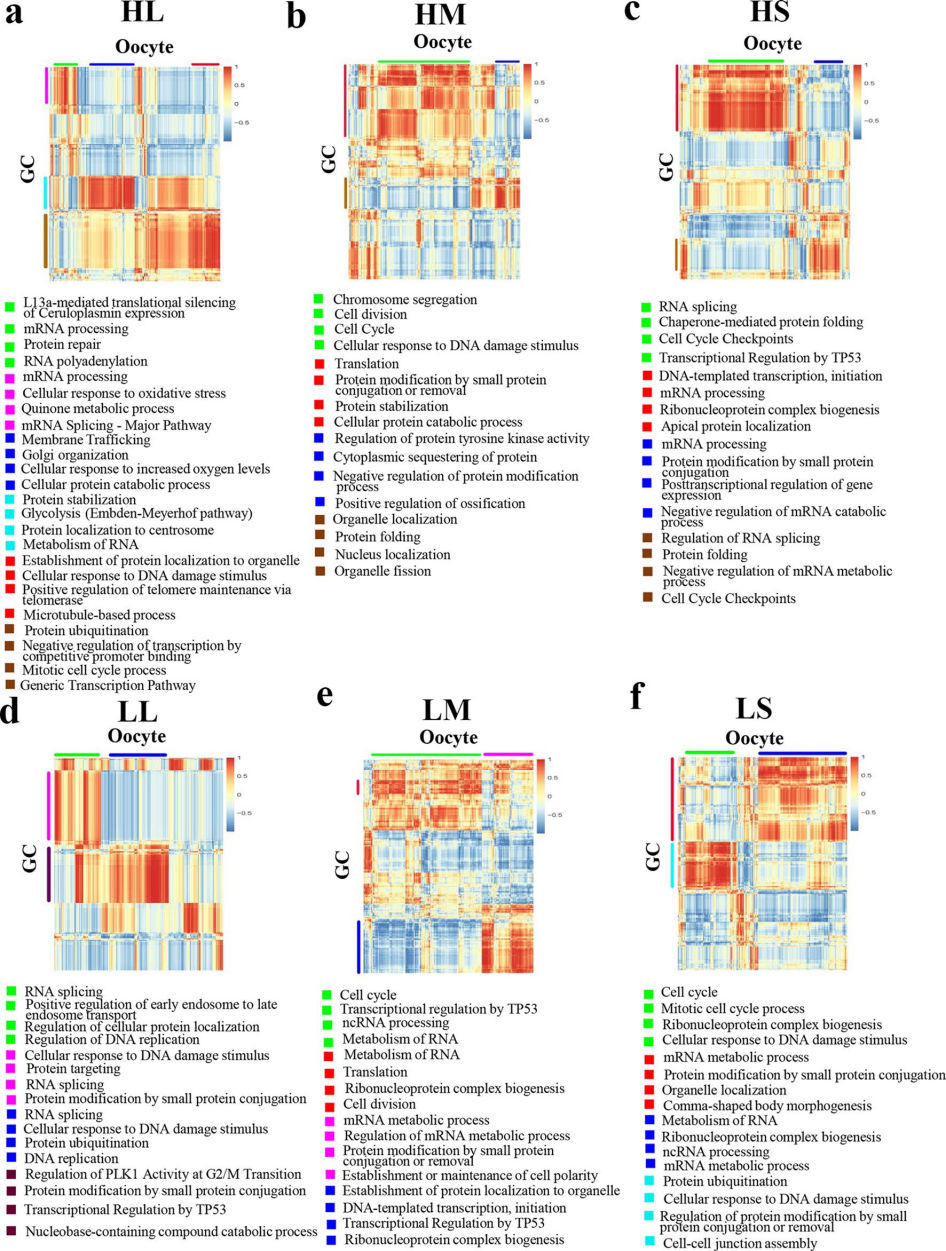
Functional co-expression between oocytes and GCs based on Spearman’s correlation coefficient. (**a**) Heatmap of genes expression between oocytes and GCs in large follicles of high fertility group; and the top four enriched functional pathways for the high correlation genes. (**b**) Heatmap of genes expression between oocytes and GCs in medium follicles of high fertility group; and the top four enriched functional pathways for the high correlation genes. (**c**) Heatmap of genes expression between oocytes and GCs in small follicles of high fertility group; and the top four enriched functional pathways for the high correlation genes. (**d**) Heatmap of genes expression between oocytes and GCs in large follicles of low fertility group; and the top four enriched functional pathways for the high correlation genes. (**e**) Heatmap of genes expression between oocytes and GCs in medium follicles of low fertility group; and the top four enriched functional pathways for the high correlation genes. (**f**) Heatmap of genes expression between oocytes and GCs in small follicles of low fertility group; and the top four enriched functional pathways for the high correlation genes. Horizontal and vertical bars next to the heatmaps annotate gene clusters with enriched biological processes.

The patterns of correlation of gene expression for different stage of follicles and different populations were present in Fig. 9. The X-axis was for oocytes and the Y-axis was for GCs. The very positively expressed genes in oocytes and GCs at different stages in the two populations were enriched to identify the functions. It was interesting that similar functional pathways were identified for oocytes and GCs with the strong positive correlation in all the stages of follicle in both high and low fertility populations. These functional pathways were related to RNA processing, protein modification (Fig. 9). And the cell cycle related pathways were enriched in the medium and small follicles not large follicles which indicated that in the early stage oocytes and GCs cell proliferation, division, or differentiation (Fig. 9b,c,e,f).

There were some pathways enriched only in high fertility population, not in low fertility population: such as “microtubule-based process”, “positive regulation of telomere maintenance via telomerase” (Fig. 9a,d); “chromosome segregation”, “cytoplasmic sequestering of protein” (Fig. 9b,e); “posttranscriptional regulation of gene expression”, “cell cycle checkpoints” (Fig. 9c,f). The data suggested that the oocytes and GCs were different with the correlation in gene expression for the two populations.

## Discussion

As the technology development, RNA-seq is a very useful tool to uncover the transcriptomic changes of oocytes and granulosa cell and to discover the interaction of different types of cells and even the mechanisms of folliculogenesis^23^. In current investigation, we explored the gene expression landscape of oocyte and GCs during different developmental stages by the tube based single cell RNA-seq. We aimed to discover the gene expression patterns in these two types of cells oocytes and GCs, the dynamic gene expression changes during development, and the interactions of genes in both sides to deeply understand the mechanism of folliculogenesis in two different populations. The data will be used to help the regulation of goat fertility (litter size).

First, we identified a set of potential genes in oocyte and GCs as the marker genes which can be used to determine the goat fertility capability and ovarian reserve ability. The data showed that *GRHPR*, *GPR84*, *CYB5A* and *ERAL1* were highly expressed in oocyte while *JUNB*, *SCN2A*, *MEGE*8, *ZEB*2, *EGR1*and *PRRC2A* were highly expressed in GCs. It has been reported that *ZEB2* has been identified as a new marker gene in human follicular GCs during the folliculogenesis^23^. Moreover, estrogen is mainly produced by aromatase in GCs. Jun, highly expressed in GCs, regulated aromatase through cAMP signaling pathway to control the estrogen level^35^. *EGR1* has been reported to be one of the target genes of fibroblast growth factors (*FGF*) in cow GCs^36^ and it can promote the apoptosis of GCs in aging mouse ovary^37^. In our another study (unpublished data) MEGE8 can be used as a marker gene in goat GCs. Many of these marker genes need to be identified in the future.

Next, we tried to explore the functional gene expression in oocytes and GCs in the different populations (high fertility group, HL; low fertility group, LL). We found much more genes are highly expressed in large and medium oocytes of HL population than that in LL population, while, more gene expressed in small oocytes of LL population than that in HL population. The enriched functional pathways in the high expressed gene in the large and medium oocytes of HL population are related to protein translation, metabolism, and transportation which are very close to the oocyte function at these stages. While the enriched functional pathways for the genes highly expressed in LL population are related to other functions. The data indicated that oocytes in HL population pose the high potential for folliculogenesis.

The gene expression profile poses the developmental trend in GCs, too. And many more genes were differentially expressed in GCs of the two populations (HL vs. LL) than that in oocytes. More genes were highly expressed in GCs of large follicles in HL group than that in LL group, while more genes were highly expressed in GCs of medium follicles in LL group than that in HL group. The enriched functional pathways in the highly expressed genes in large and medium follicular GCs were related to cell cycle and proliferation which is close to the function of GCs at these stages. The data suggested that the expression of genes in GCs were correlated to the oocyte for the folliculogenesis which may reflect the fertility difference in these populations.

It has been reported recently ligands and receptors mediate the regulatory signaling between follicular oocytes and GCs during folliculogenesis (Biase and Kimble 2018). And the transcriptome profile of oocytes^18–21^ and GCs^7–17^ can reflect the developmental potential of oocyte to be successfully fertilized to develop to be embryo. Moreover, ligand-receptor pairs can tune the signaling for the regulation of gene expression (Biase and Kimble 2018). In current investigation, we found that ligand-receptor pairs played important roles in goat folliculogenesis. Notch signaling pathway has been reported to be one of the most important pathways in folliculogenesis^38^. *DLL3*, a ligand in Notch pathway, was highly expressed in oocytes, while another ligand in Notch pathways *JAG2* was highly expressed in GCs compare with DLL3 and JAG1. The receptors in Notch pathway *NOTCH2* and *NOTCH3* were highly expressed in GCs while the target gene *HES1* was highly expressed in oocytes. The transforming growth factor-β (*TGF*-β) superfamily plays vital roles in the tuning folliculogenesis^39^. The important ligands in *TGF*-β pathway were highly expressed in oocytes while the receptor *BMPR1b* was highly expressed in GCs. The target gene *ID3* was highly expressed in oocytes while *ID4* was highly expressed in GCs. Moreover, we found ligand-receptor pairs were differentially expressed in the oocytes and GCs of HL and LL at different developmental stages. The data suggested that the gene expression levels are differentially regulated by ligand-receptors in HL and LL population during folliculogenesis.

Furthermore, the correlations of the gene expression in oocytes and GCs at different stages in the two populations HL and LL were different, too. It has been reported that the association between the transcript levels of genes expressed in oocytes and GCs play important roles in follicogenesis, too^24^. Our data matched this notion very well.

## Conclusions

In summary, in the current investigation we found many marker genes differentially expressed in oocytes or GCs during folliculogenesis. The ligand-receptor pairs in oocyte-GCs are involved in the regulation of the gene expression profile in two fertility population HL and LL. At the same time, the gene expression pattern was correlated well in oocyte and GCs in HL or LL. All the data reflect the gene expression landscape in oocytes and GCs which is correlated well with the fertility capability.

## Supplementary Information

Supplementary Information 1.

Supplementary Information 2.

Supplementary Information 3.

## Data Availability

The 10× sequencing raw data are deposited in NCBI’s Gene Expression Omnibus under accession number: GSE135897 and GSE136005.

## Abbreviations

GCs: Granulosa cells
IACUC: Institutional Animal Care and Use Committee
HL: High fertility
LL: Low fertility
HSO: Small follicular oocyte in high fertility group
HMO: Medium follicular oocyte in high fertility group
HLO: Large follicular oocyte in high fertility group
LSO: Small follicular oocyte in low fertility group
LMO: Medium follicular oocyte in low fertility group
LLO: Large follicular oocyte in low fertility group
HSG: Small follicular granulosa cells in high fertility group
HMG: Medium follicular granulosa cells in high fertility group
HLG: Large follicular granulosa cells in high fertility group
LSG: Small follicular granulosa cells in low fertility group
LMG: Medium follicular granulosa cells in low fertility group
LLG: Large follicular granulosa cells in low fertility group
FPKM: Fragments Per Kilobase of exon model per Million mapped fragments
PPI: Protein–protein interactions
q-RT-PCR: Quantitative reverse transcriptional PCR
IHF: Immunofluorescence staining
PBS: Phosphate buffer saline
DAPI: 4′,6-Diamidino-2-phenylindole

## References

[1] Conti M, Hsieh M, Zamah AM, Oh JS (2012). Novel signaling mechanisms in the ovary during oocyte maturation and ovulation. Mol. Cell Endocrinol..

[2] Amireault P, Dube F (2005). Intracellular cAMP and calcium signaling by serotonin in mouse cumulus-oocyte complexes. Mol. Pharmacol..

[3] Gilchrist RB, Ritter LJ, Armstrong DT (2004). Oocyte-somatic cell interactions during follicle development in mammals. Anim. Reprod. Sci..

[4] Wigglesworth K, Lee KB, O'Brien MJ, Peng J, Matzuk MM, Eppig JJ (2013). Bidirectional communication between oocytes and ovarian follicular somatic cells is required for meiotic arrest of mammalian oocytes. Proc. Natl. Acad. Sci. U.S.A..

[5] De la Fuente R, Eppig JJ (2001). Transcriptional activity of the mouse oocyte genome: Companion granulosa cells modulate transcription and chromatin remodeling. Dev. Biol..

[6] Macaulay AD, Gilbert I, Scantland S, Fournier E, Ashkar F, Bastien A, Saadi HA, Gagne D, Sirard MA, Khandjian EW (2016). Cumulus cell transcripts transit to the bovine oocyte in preparation for maturation. Biol. Reprod..

[7] Dieci C, Lodde V, Labreque R, Dufort I, Tessaro I, Sirard MA, Luciano AM (2016). Differences in cumulus cell gene expression indicate the benefit of a pre-maturation step to improve in-vitro bovine embryo production. Mol. Hum. Reprod..

[8] Molinari E, Bar H, Pyle AM, Patrizio P (2016). Transcriptome analysis of human cumulus cells reveals hypoxia as the main determinant of follicular senescence. Mol. Hum. Reprod..

[9] Assidi M, Montag M, Sirard MA (2015). Use of both cumulus cells' transcriptomic markers and zona pellucida birefringence to select developmentally competent oocytes in human assisted reproductive technologies. BMC Genomics.

[10] Bunel A, Nivet AL, Blondin P, Vigneault C, Richard FJ, Sirard MA (2014). Cumulus cell gene expression associated with pre-ovulatory acquisition of developmental competence in bovine oocytes. Reprod. Fertil. Dev..

[11] Bunel A, Jorssen EP, Merckx E, Leroy JL, Bols PE, Sirard MA (2015). Individual bovine in vitro embryo production and cumulus cell transcriptomic analysis to distinguish cumulus-oocyte complexes with high or low developmental potential. Theriogenology.

[12] Macaulay AD, Gilbert I, Caballero J, Barreto R, Fournier E, Tossou P, Sirard MA, Clarke HJ, Khandjian EW, Richard FJ (2014). The gametic synapse: RNA transfer to the bovine oocyte. Biol. Reprod..

[13] Nivet AL, Vigneault C, Blondin P, Sirard MA (2013). Changes in granulosa cells' gene expression associated with increased oocyte competence in bovine. Reproduction.

[14] Vigone G, Merico V, Prigione A, Mulas F, Sacchi L, Gabetta M, Bellazzi R, Redi CA, Mazzini G, Adjaye J (2013). Transcriptome based identification of mouse cumulus cell markers that predict the developmental competence of their enclosed antral oocytes. BMC Genomics.

[15] Tesfaye D, Ghanem N, Carter F, Fair T, Sirard MA, Hoelker M, Schellander K, Lonergan P (2009). Gene expression profile of cumulus cells derived from cumulus–oocyte complexes matured either in vivo or in vitro. Reprod. Fertil. Dev..

[16] Jiang JY, Xiong H, Cao M, Xia X, Sirard MA, Tsang BK (2010). Mural granulosa cell gene expression associated with oocyte developmental competence. J. Ovarian Res..

[17] Biase FH, Fonseca Merighe GK, Santos Biase WK, Martelli L, Meirelles FV (2008). Global poly(A) mRNA expression profile measured in individual bovine oocytes and cleavage embryos. Zygote.

[18] Biase FH (2017). Oocyte developmental competence: Insights from cross-species differential gene expression and human oocyte-specific functional gene networks. OMICS.

[19] Biase FH, Everts RE, Oliveira R, Santos-Biase WK, Fonseca Merighe GK, Smith LC, Martelli L, Lewin H, Meirelles FV (2014). Messenger RNAs in metaphase II oocytes correlate with successful embryo development to the blastocyst stage. Zygote.

[20] Biase FH, Martelli L, Puga R, Giuliatti S, Santos-Biase WKF, Fonseca Merighe GK, Meirelles FV (2010). Messenger RNA expression of Pabpnl1 and Mbd3l2 genes in oocytes and cleavage embryos. Fertil. Steril..

[21] Biase FH, Martelli L, Merighe GK, Santos Biase WK, Miranda M, Smith LC, Meirelles FV (2009). A retrospective model of oocyte competence: Global mRNA and housekeeping transcripts are not associated with in vitro developmental outcome. Zygote.

[22] Sanchez F, Smitz J (2012). Molecular control of oogenesis. Biochim. Biophys. Acta.

[23] Zhang Y, Yan Z, Qin Q, Nisenblat V, Chang H, Yu Y, Wang T, Lu C, Yang M, Yang S, Yao Y, Zhu X, Xia X, Dang Y, Ren Y, Yuan P, Li R, Liu P, Guo H, Han J, He H, Zhang K, Wang Y, Wu Y, Li M, Qiao J, Yan J, Yan L (2018). Transcriptome landscape of human folliculogenesis reveals oocyte and granulosa cell interactions. Mol. Cell..

[24] Biase FH, Kimble KM (2018). Functional signaling and gene regulatory networks between the oocyte and the surrounding cumulus cells. BMC Genomics.

[25] Miao X, Luo Q, Qin X (2016). Genome-wide transcriptome analysis in the ovaries of two goats identifies differentially expressed genes related to fecundity. Gene.

[26] Dong Y, Xie M, Jiang Y, Xiao N, Du X, Zhang W, Tosser-Klopp G, Wang J, Yang S, Liang J, Chen W, Chen J, Zeng P (2013). Sequencing and automated whole-genome optical mapping of the genome of a domestic goat (Capra hircus). Nat. Biotechnol..

[27] Ko JH, Lee CS, Kim KH, Pang MG, Koo JS, Fang N, Koo DB, Oh KB, Youn WS, Zheng GD, Park JS, Kim SJ, Han YM, Choi IY, Lim J, Shin ST, Jin SW, Lee KK, Yoo OJ (2000). Production of biologically active human granulocyte colony stimulating factor in the milk of transgenic goat. Transgenic Res..

[28] El-Tarabany MS, Zaglool AW, El-Tarabany AA, Awad A (2017). Association analysis of polymorphism in KiSS1 gene with reproductive traits in goats. Anim. Reprod. Sci..

[29] Shabir M, Ganai TAS, Misra SS, Shah R, Ahmad T (2013). Polymorphism study of growth differentiation factor 9B (GDF9B) gene and its association with reproductive traits in sheep. Gene.

[30] Zhao Y, Zhang P, Ge W, Feng Y, Li L, Sun Z, Zhang H, Shen W (2020). Alginate oligosaccharides improve germ cell development and testicular microenvironment to rescue busulfan disrupted spermatogenesis. Theranostics..

[31] Yu S, Zhao Y, Lai F, Chu M, Hao Y, Feng Y (2017). LncRNA as ceRNAs may be involved in lactation process. Oncotarget.

[32] Skelly DA, Squiers GT, McLellan MA, Bolisetty MT, Robson P, Rosenthal NA, Pinto AR (2018). Single-cell transcriptional profiling reveals cellular diversity and intercommunication in the mouse heart. Cell Rep..

[33] Ramilowski JA, Goldberg T, Harshbarger J, Kloppmann E, Lizio M, Satagopam VP, Itoh M, Kawaji H, Carninci P, Rost B, Forrest AR (2015). A draft network of ligand–receptor-mediated multicellular signaling in human. Nat. Commun..

[34] Kidder GM, Vanderhyden BC (2010). Bidirectional communication between oocytes and follicle cells: Ensuring oocyte developmental competence. Can. J. Physiol. Pharm..

[35] Ghosh S, Wu Y, Li R, Hu Y (2005). Jun proteins modulate the ovary-specific promoter of aromatase gene in ovarian granulosa cells via a cAMP-responsive element. Oncogene.

[36] Han P, Guerrero-Netro H, Estienne A, Cao B, Price CA (2017). Regulation and action of early growth response 1 in bovine granulosa cells. Reproduction.

[37] Yuan S, Wen J, Cheng J, Shen W, Zhou S, Yan W, Shen L, Luo A, Wang S (2016). Age-associated up-regulation of EGR1 promotes granulosa cell apoptosis during follicle atresia in mice through the NF-κB pathway. Cell Cycle.

[38] Prasasya RD, Mayo KE (2018). Notch signaling regulates differentiation and steroidogenesis in female mouse ovarian granulosa cells. Endocrinology.

[39] Chang H, Qiao J, Leung PKC (2016). Oocyte–somatic cell interactions in the human ovary-novel role of bone morphogenetic proteins and growth differentiation factors. Hum. Reprod. Updat..

