# Supplementary material for: Transcriptome profile of goat folliculogenesis reveals the interaction of oocyte and granulosa cell in correlation with different fertility population
Source: Sci Rep. 2021 Aug 3;11:15698. doi: 10.1038/s41598-021-95215-z (PMC8333342; doi:10.1038/s41598-021-95215-z)
Supplement: Supplementary file 1 — Supplementary Information 1. [file 41598_2021_95215_MOESM1_ESM.pdf]

Supplemental information

Fig. S1

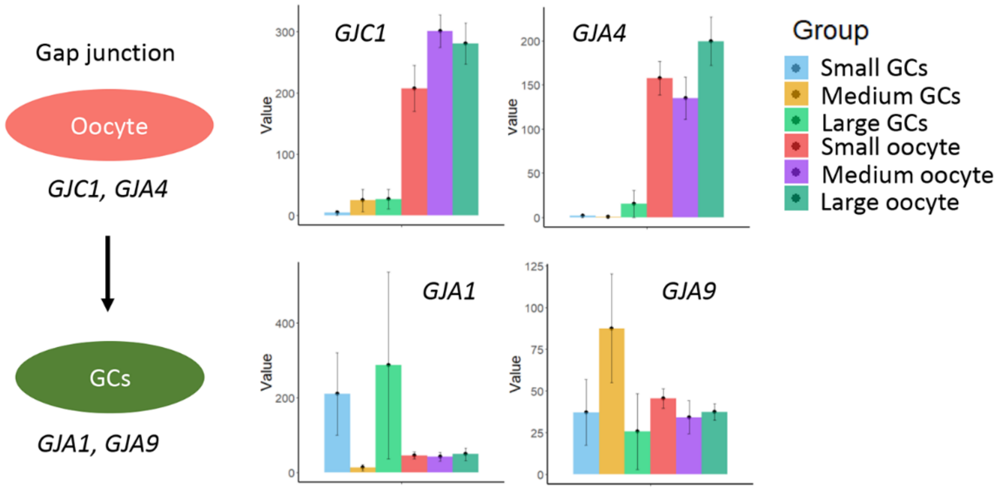

Fig. S1. Interaction of genes in cell junction pathways.

Fig. S2

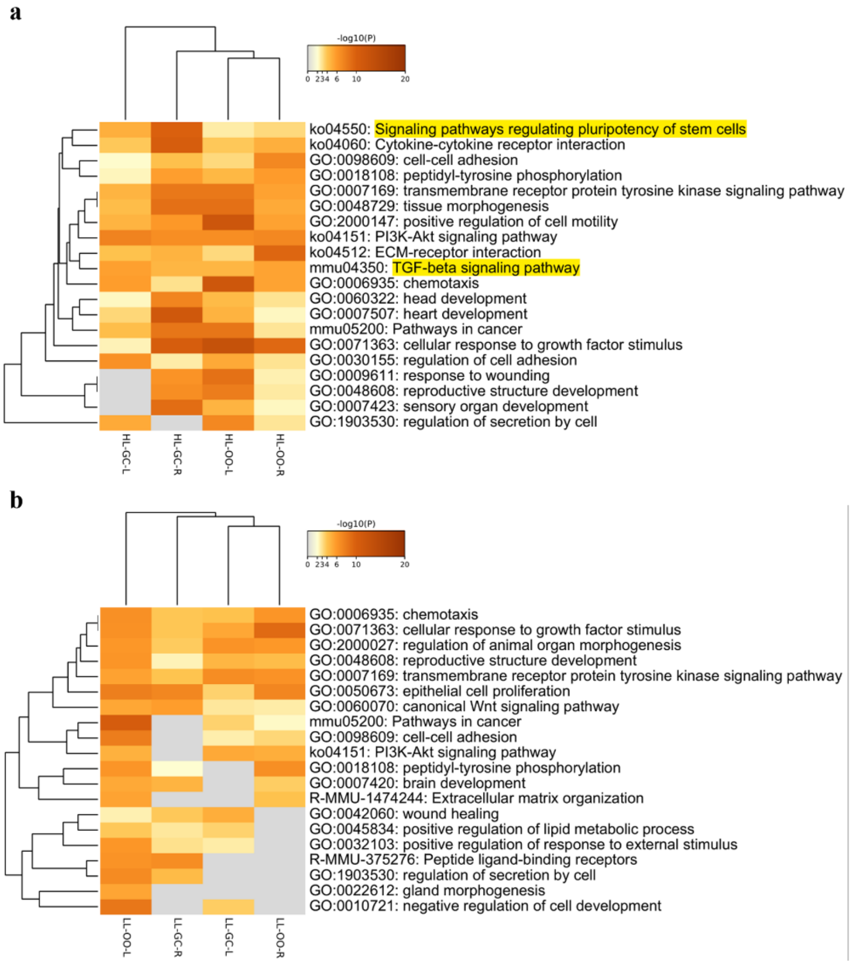

Fig. S2. Functional enrichment of genes in the ligand-receptor pairs. (**a**) Enriched pathways of ligand (L) receptor (R) pairs in large follicular oocyte and GCs of high fertility group. (**b**) Enriched pathways of ligand (L) receptor (R) pairs in large follicular oocyte and GCs of low fertility group.

Fig. S3

a

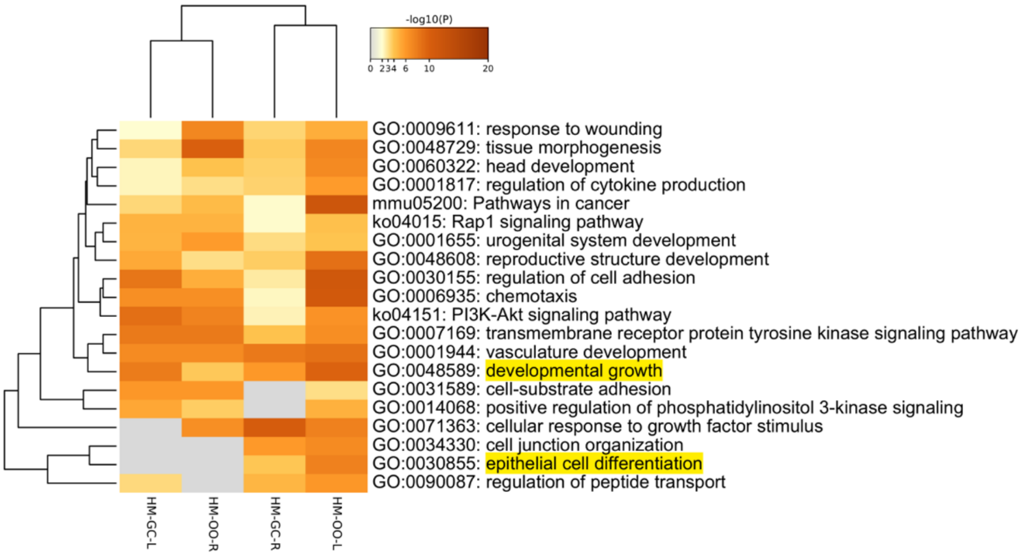

b

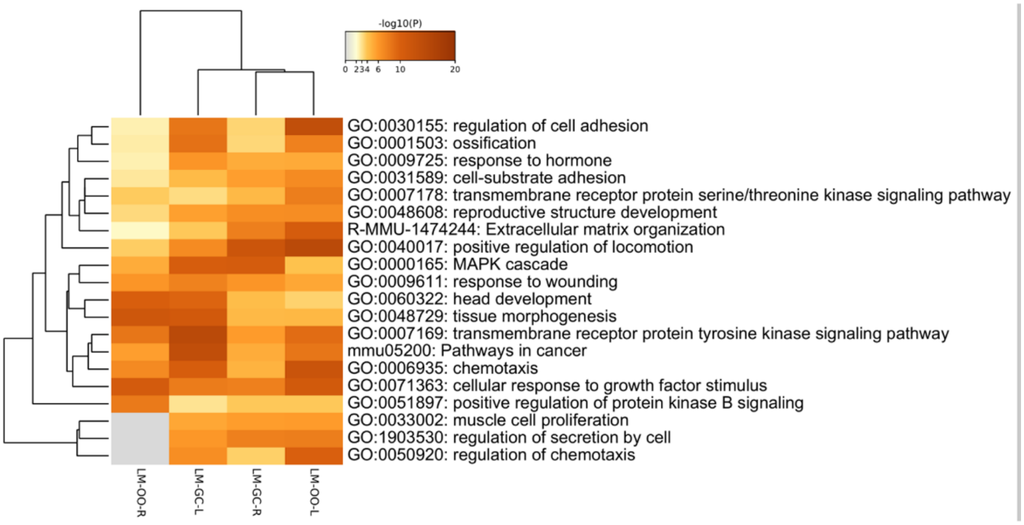

Fig. S3. Functional enrichment of genes in the ligand-receptor pairs. (**a**) Enriched pathways of ligand (L) receptor (R) pairs in medium follicular oocyte and GCs of high fertility group. (**b**) Enriched pathways of ligand (L) receptor (R) pairs in medium follicular oocyte and GCs of low fertility group.

Fig. S4

a

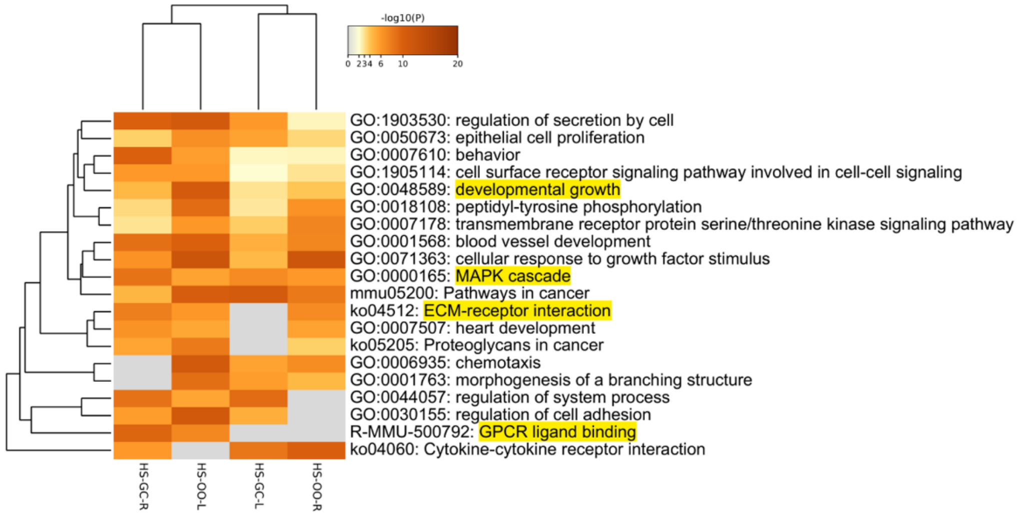

b

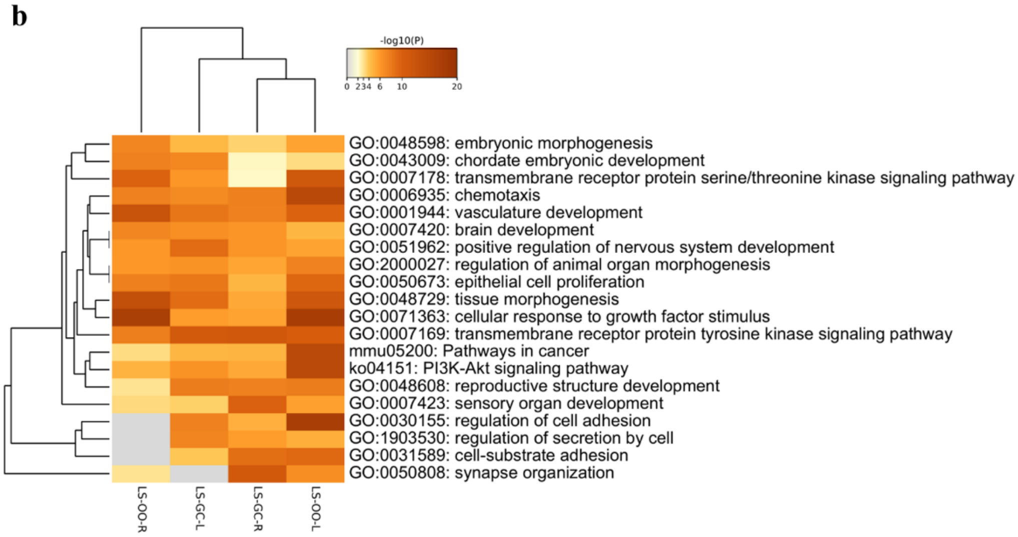

Fig. S4. Functional enrichment of genes in the ligand-receptor pairs. (**a**) Enriched pathways of ligand (L) receptor (R) pairs in small follicular oocyte and GCs of high fertility group. (**b**) Enriched pathways of ligand (L) receptor (R) pairs in small follicular oocyte and GCs of low fertility group.

Table S1. Primer sets.

NEFM (goat)Forward	AGGAAGAGGAGGCTGAAGAA
NEFM (goat)Reverse	TCTTTAAGTTCAGGCGCAGTAG
LMX1A (goat)Forward	TGTGGTTCAGGTGTGGTTC
LMX1A (goat)Reverse	CTGGGTGTTCTGCTGATCTT
DACT1 (goat)Forward	ACCGTCAAGACAGACACTAAC
DACT1(goat)Reverse	GGACTAGGCTCAGAATGTAACC
MCPH1(goat)Forward	GTGTACAGTCCGACAGGTAAAG
MCPH1(goat)Reverse	CTGTTCAATCATCTGGGAGGAG
CD38 (goat) Forward	CTAGGGACCAGTTCTGGTTAAG
CD38 (goat) Reverse	GTGGATTAGCAGGGACCTATTT
PNPLA3 (goat)Forward	CCTGTGATGTACGCAGAATGA
PNPLA3 (goat)Reverse	CACACCAAGAGTAGCTTCCAA
DKKL1 (goat)Forward	GGAACTACCACCAAGAAGAGAAC
DKKL1 (goat)Reverse	ATCACCACCTCTCCTGTCTT
PLCZ1 (goat)Forward	GATAAGCATGGGCAGGTAGAG
PLCZ1 (goat)Reverse	AAATCACCCACTGGTTCTGG
STRBP (goat)Forward	GGCTGTAGACACCTATTCCAAG
STRBP (goat)Reverse	CCAGCTCCAAGTCCATATCATC
INHBB (goat)Forward	CTGGACAATGCACACGTAGA
INHBB (goat)Reverse	TGTGGCTGAGCTGCTATTT

Table S2. RNA-seq data for sequence counts.

Table S3. Mapping information.
